# Loss of UCP2 Attenuates Mitochondrial Dysfunction without Altering ROS Production and Uncoupling Activity

**DOI:** 10.1371/journal.pgen.1004385

**Published:** 2014-06-19

**Authors:** Alexandra Kukat, Sukru Anil Dogan, Daniel Edgar, Arnaud Mourier, Christoph Jacoby, Priyanka Maiti, Jan Mauer, Christina Becker, Katharina Senft, Rolf Wibom, Alexei P. Kudin, Kjell Hultenby, Ulrich Flögel, Stephan Rosenkranz, Daniel Ricquier, Wolfram S. Kunz, Aleksandra Trifunovic

**Affiliations:** 1 Cologne Excellence Cluster on Cellular Stress Responses in Aging-Associated Diseases (CECAD) and Institute for Mitochondrial Diseases and Aging, Medical Faculty, University of Cologne, Cologne, Germany; 2 Department of Laboratory Medicine, Karolinska Institutet, Stockholm, Sweden; 3 Max Planck Institute for Biology of Aging, Cologne, Germany; 4 Department of Cardiovascular Physiology, Heinrich-Heine-University, Düsseldorf, Germany; 5 Max Planck Institute for Neurological Research, Cologne, Germany; 6 Department of Epileptology, University of Bonn, Bonn, Germany; 7 Department III of Internal Medicine, University of Cologne, Cologne, Germany; 8 Cologne Cardiovascular Research Center (CCRC) and Center for Molecular Medicine Cologne, University of Cologne, Cologne, Germany; 9 University Paris Descartes, Faculty of Medicine, CNRS FRE3210, Paris, France; Max Planck Institute for Biology of Ageing, Germany

## Abstract

Although mitochondrial dysfunction is often accompanied by excessive reactive oxygen species (ROS) production, we previously showed that an increase in random somatic mtDNA mutations does not result in increased oxidative stress. Normal levels of ROS and oxidative stress could also be a result of an active compensatory mechanism such as a mild increase in proton leak. Uncoupling protein 2 (UCP2) was proposed to play such a role in many physiological situations. However, we show that upregulation of UCP2 in mtDNA mutator mice is not associated with altered proton leak kinetics or ROS production, challenging the current view on the role of UCP2 in energy metabolism. Instead, our results argue that high UCP2 levels allow better utilization of fatty acid oxidation resulting in a beneficial effect on mitochondrial function in heart, postponing systemic lactic acidosis and resulting in longer lifespan in these mice. This study proposes a novel mechanism for an adaptive response to mitochondrial cardiomyopathy that links changes in metabolism to amelioration of respiratory chain deficiency and longer lifespan.

## Author Summary

Mitochondria produce the majority of the energy needed for numerous cell functions through oxidative phosphorylation. However, this comes with the cost in the form of potentially harmful reactive oxygen species (ROS) that could damage all kinds of biological macromolecules. Changes in mitochondrial membrane potential through mild uncoupling could alter ROS production in the cell (“uncoupling to survive”). Mitochondrial uncoupling proteins (UCPs) are believed to play a central role in this process. We detected increased amounts of UCP2 in mtDNA mutator mice, a model for premature aging. Depletion of UCP2 in mtDNA mutator mice led to further shortening of the lifespan with earlier signs of mitochondrial cardiomyopathy accompanied with high systemic lactic acidosis, often used as a marker of mitochondrial diseases. Remarkably, our results demonstrate that the presence of UCP2 wields beneficial effect on respiratory deficient mitochondria without affecting ROS production or uncoupling. Instead, UCP2 protein seems to mediate a valuable upregulation of fatty acid metabolism detected in mtDNA mutator hearts. Our results provide a novel mechanism of adaptation of mitochondria to respiratory deficiency mediated by UCP2 that clearly argues against the “uncoupling to survive” theory.

## Introduction

Mitochondria are organelles found in almost every eukaryotic cell. They produce the bulk of cellular energy in the form of ATP, which is required for numerous processes in the cell. Still, mitochondrial energy production comes with a cost, the generation of reactive oxygen species (ROS) that is linked to the development of different pathologies and is proposed to contribute to aging [1]. The mitochondrial theory of aging proposes that an age-driven accumulation of mtDNA mutations will compromise electron transport leading to an increase in ROS production [1]. We challenged this theory by showing that increased levels of random mtDNA mutations lead to the development of premature aging phenotypes in mtDNA mutator mice, without affecting ROS production or increasing oxidative stress [2], [3]. Our result argues against a direct link between mtDNA mutations and increased ROS production, but can also point out to the consequence of an active compensatory mechanism. A mild uncoupling of oxidative phosphorylation, leading to decreased mitochondrial ATP production could be sufficient to reduce ROS generation in the cell [4]. The “uncoupling to survive” theory further suggests that mitochondrial inefficiency mediated by partial uncoupling could have a beneficial effect on aging [4]. Uncoupling proteins (UCPs) are proposed to have a central role in this process (for review see [5]).

The mitochondrial uncoupling proteins (UCPs) are located in the mitochondrial inner membrane where they could act as regulated protonophores. The term “uncoupling protein” was originally used for UCP1, a brown fat specific proton carrier that dissipates the proton gradient as heat [6]. It was anticipated that UCP2 and UCP3 lead to a moderate uncoupling that is believed to modulate the ATP/ADP ratio for signalling purposes, but their precise function in normal cellular physiology is still unclear [7]. These proteins are found in much lesser amounts than UCP1 and might be involved in the proton conductance only upon activation leading to the conclusion that they are not involved in the adaptive response to cold [7]. Emerging evidence suggests that UCP2 plays a positive physiological role by regulating mitochondrial biogenesis, substrate utilization, and ROS elimination; thereby, provides neuroprotective [8] and possibly anti-aging effects [9]. Nevertheless, several studies indicated that UCP2 could have deleterious effects on cellular function, like in the development of insulin resistance and the pathogenesis of type 2 diabetes mellitus [10].

In this study we detected increased amounts of UCP2 in multiple tissues of mtDNA mutator mice. We show that UCP2 has a protective role against molecular changes leading to a progressive cardiomyopathy and complete loss of this protein leads to further shortening of the lifespan in mtDNA mutator mice. However, UCP2 exercises its beneficial effect without affecting ROS production or modulating proton leak kinetics in heart. Instead, we provide evidence for a novel mechanism by which increased UCP2 levels modulate cardiac metabolism to maintain proficient energy production, challenged by progressive respiratory deficiency.

## Results

### UCP2 levels are increased in mtDNA mutator mice and depletion of this protein leads to further lifespan shortening

**Figure 1:**
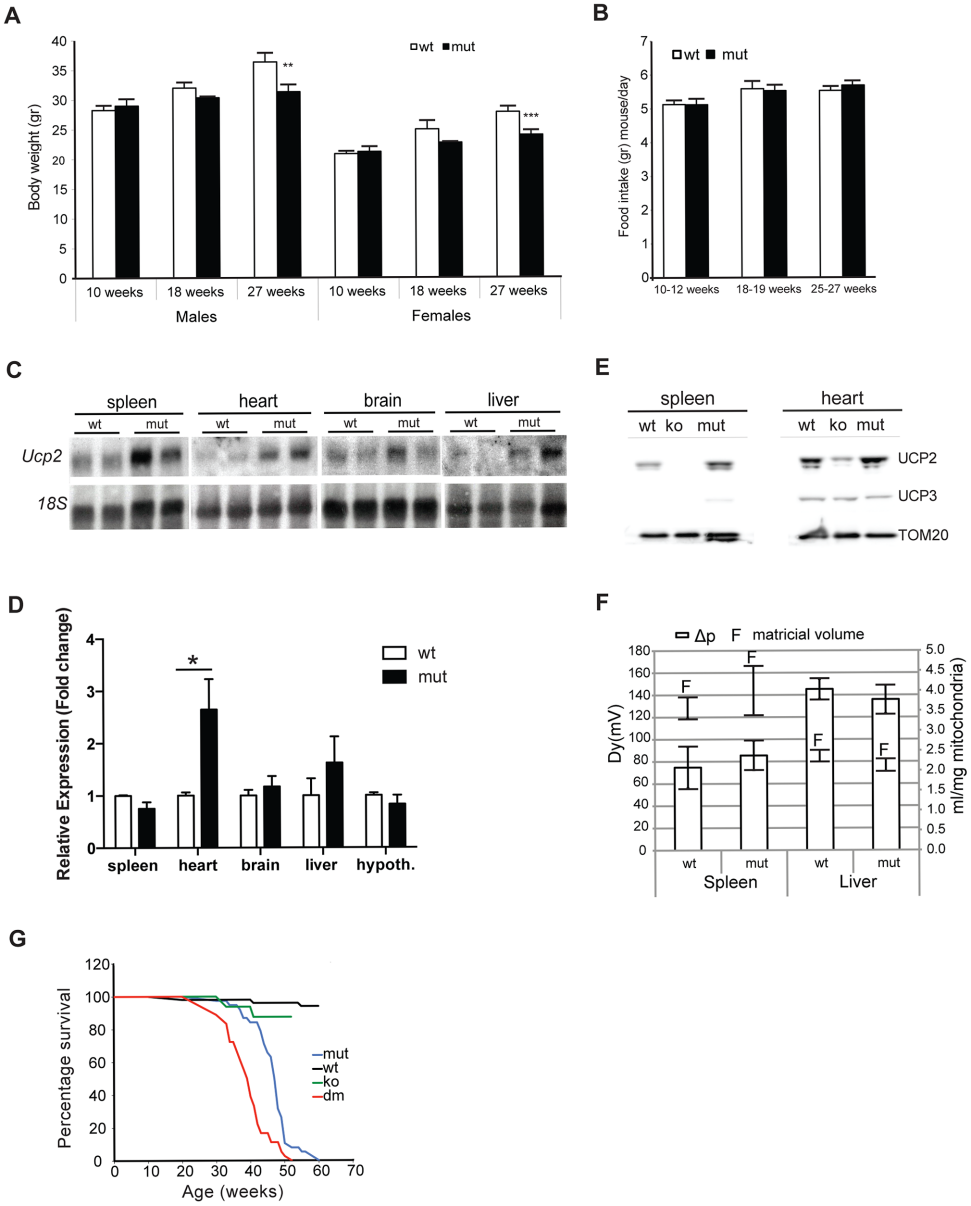
MtDNA mutator mice have lower body mass and upregulated UCP2 levels. Body weight (A) and daily food consumption (B) in mtDNA mutator (mut) mice in comparison to littermate controls (wt) (n = 11). (C) Northern blot analyses of *Ucp2* levels in spleen, heart, brain and liver. (D) Fold change of *Ucp2* transcript levels in spleen, heart, brain, liver and hypothalamus for wt and mut (n = 4). (E) Western blot analyses of UCP2 in spleen and heart mitochondria of wt, mut and UCP2-deficient (ko) mice. (F) Proton motive force (bars) and mitochondrial matrix volume (lines) in liver and spleen mitochondria (n = 3). (G) Mean lifespan of wt, mut, ko and dm mice. All analyses were performed on 25-week-old mice. Bars indicate mean level ± standard error of the mean (S.E.M.). Asterisks indicate level of statistical significance (*p<0.05 **p<0.01 ***p<0.001, Student's *t*-test).

One of the first symptoms of premature aging in mtDNA mutator mice is cessation in weight gain around 18–25 weeks of age, followed by a progressive loss of body mass with increasing age [2]. Indeed, we detected a significant difference in the body weight in male and female mtDNA mutator mice already at 18 weeks of age that was even more pronounced at 25–27 weeks of age (Figure 1A). Both individually and group-housed mtDNA mutator mice maintained on a regular chow diet showed normal daily food intake at different time points that included measuring food consumption over periods of several weeks (2–3 weeks) (Figure 1B). Although we cannot completely exclude the possibility that mtDNA mutator mice had decreased food intake at some point between these measurements, we believe this to be unlikely. During our early screening for changes in gene expression, we noticed that the expression of *Ucp2* is increased in some tissues of mtDNA mutator mice, most prominently in heart (Figure 1C and 1D). The increase in *Ucp2* mRNA levels was mirrored by an increase in the UCP2, but not UCP3, protein levels in heart and spleen (Figure 1E). Interestingly, we failed to observe an increase in *Ucp2* transcript levels in spleen by quantitative real-time PCR, although we observed clear upregulation of protein levels (Figure 1D and 1E, respectively). This could be the consequence of increased UCP2 stability in spleen.

Inefficient energy use caused by the uncoupling of mitochondrial respiration could lead to the observed imbalance between food intake and energy expenditure in mtDNA mutator mice. An increased energy expenditure and lower body mass were observed in mice overexpressing human UCP3 protein specifically in skeletal muscle [11].

In order to assess whether the UCP2 upregulation leads to an increased proton leak, we measured proton motive force (Δp) in both liver and spleen mitochondria (Figure 1F). These two tissues represent the real opposites when it comes to the UCP2 levels: while splenocytes contain the highest level of UCP2, the transcripts detected in liver preps originate from the Kupffer cells, which are resident liver macrophages, as it was shown that hepatocytes do not express UCP2 [12]. Despite the upregulation of UCP2 levels in mtDNA mutator mitochondria isolated from spleen, we could not observe a difference in the Δp between the two genotypes indicating that UCP2 does not contribute significantly to the proton leak in these conditions (Figure 1F). We also show that mitochondrial matricial volume, on which Δp is dependent, was not significantly changed (Figure 1F). Our results demonstrate that liver mitochondria have a higher proton motive force than spleen mitochondria (Figure 1F), in agreement with a previous report showing that spleen mitochondria have the highest and liver the lowest proton leak [13].

To further address the specific role of UCP2 in mitochondrial dysfunction caused by an increased mtDNA mutation load, we have crossed mtDNA mutator mice with UCP2-deficient mice (KO), producing *PolgA^mut/mut^; Ucp2^−/−^* double mutant mice (hereafter DM mice). The mean lifespan of mtDNA mutator mice was on average 14% shorter in the absence of UCP2 (DM – 39.1±4.4 weeks v. mtDNA mutator 46.4±5.3 weeks), indicating that UCP2 overexpression provides some form of beneficial adaptive change (Figure 1G). It was previously shown that UCP2-deficient mice have a significantly shorter lifespan than wild type controls [14], therefore, at this point it is difficult to distinguish if the shorter lifespan reflects a specific interaction or just an additive effect of UCP2 deficiency in mtDNA mutator mice.

### mtDNA mutator mice display a fasting-like phenotype that is not affected by the loss of UCP2

**Figure 2:**
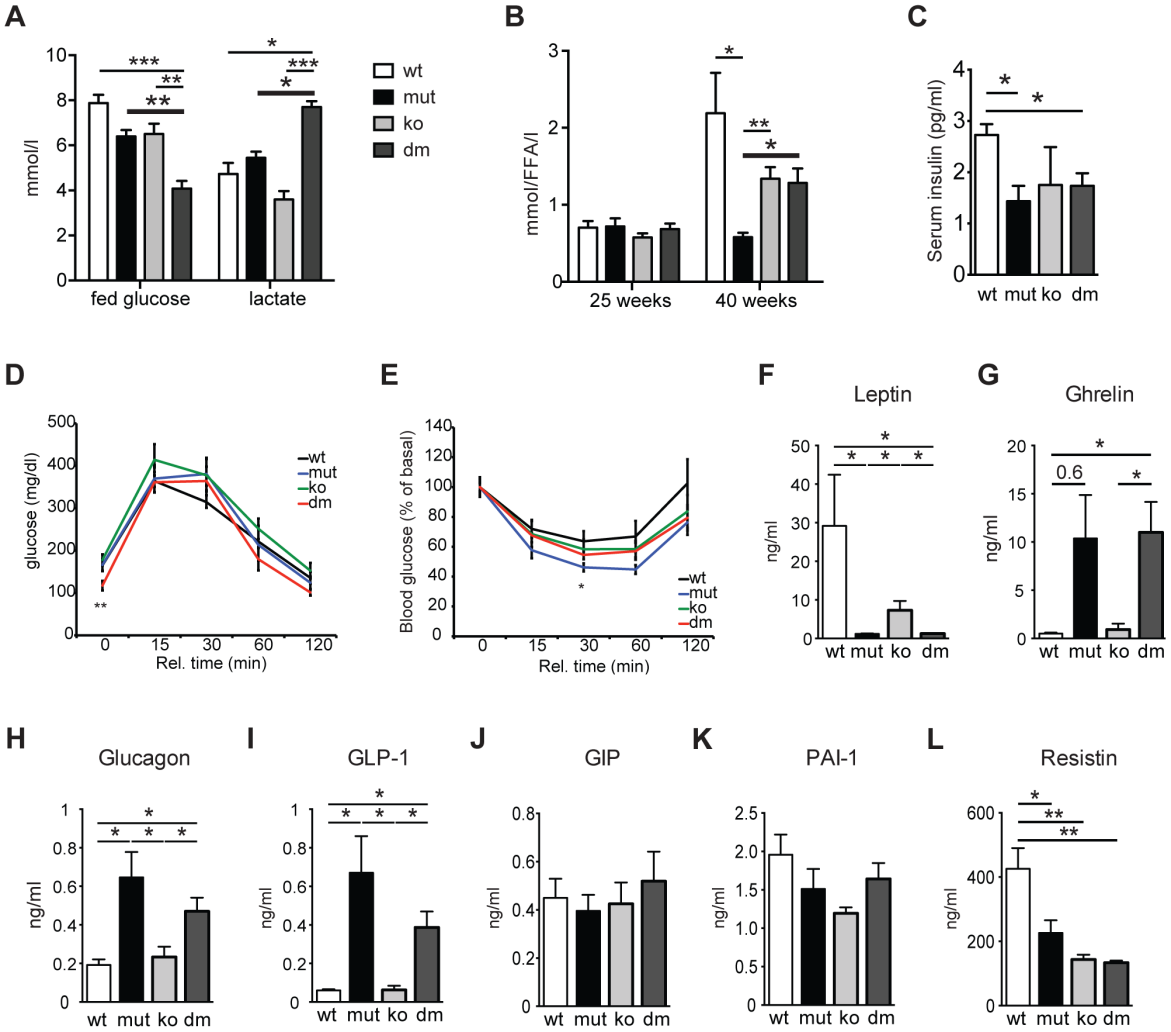
Characterization of blood metabolites in wild type (wt), mtDNA mutator (mut), UCP2-deficient wild type (ko) and mtDNA mutator (dm) mice. (A) Blood glucose and lactate concentrations at 25 weeks of age. (n = 6). (B) Circulating free fatty acid levels in 25- and 40-week-old animals. (n = 5–6). (C) Serum insulin levels of 30-week-old mice. (D) Glucose tolerance test in 30-week-old mice. After 16 hours of starvation period, mice were injected with 2g/kg body weight glucose and clearance was measured after 0, 15, 30, 60 and 120 minutes (n = 7). (E) Insulin tolerance test of 30-week-old random fed mice. Mice were injected with 0.75 U/kg body weight of insulin and glucose levels were measured after 0, 15, 30 60 and 120 minutes (n = 7). (F–L) Analyses of metabolic markers in the serum: (F) Leptin; (G) Ghrelin; (H) Glucagon; (I) Glucagon-like peptide 1 (GLP-1); (J) Glucose-dependent insulinotropic polypeptide (GIP); (K) Plasminogen activator inhibitor-1 (PAI-1); (L) Resistin. All measurements were performed in 30-week-old mice. (n = 4). Bars indicate mean levels ± standard error of the mean (S.E.M.). Statistically significant differences between mut and dm are presented with thick lines. Asterisks indicate level of statistical significance (*p<0.05; **p<0.005; ***p<0.001, Student's *t*-test).

Recent advancements in the understanding of cellular glucose and lipid metabolism identified UCP2 as a critical regulator of cellular fuel utilization and whole body glucose and lipid metabolism (for review see [15]). Therefore, we investigated the impact of UCP2 depletion on energy metabolism in mtDNA mutator mice. We observed a significant reduction in body weight in both, mtDNA mutator and DM mice at 20 weeks of age (Figure S1A – S1C). Interestingly, the oxygen consumption, energy expenditure and respiratory exchange ratio (RER) were increased only in DM mice (Figure S1D - S1G). This was accompanied by a twofold decrease in blood glucose levels and a high increase in circulating lactate levels exclusively in DM mice (Figure 2A). Taken together these data suggest that the loss of UCP2 promotes preferential usage of glucose as energy source in DM mice. Indeed, UCP2 has previously been shown to promote mitochondrial FAO while limiting mitochondrial catabolism of pyruvate originating from glucose [16]. In agreement with this, mtDNA mutator mice failed to increase circulating free fatty acid (FFA) levels at 40 weeks of age probably owing to exhaustion of lipid stores as a result of increased FAO enabled by high UCP2 upregulation (Figure 2B).

UCP2 was shown to be a negative regulator of glucose-stimulated insulin secretion (GSIS) through control of ROS production that plays an important signaling role in insulin secretion in pancreatic β-cells [10], [17]. We detected a small but significant decrease in serum insulin levels in both mtDNA mutator and DM animals at 30 weeks of age (Figure 2C). Although an initial report using mice on a mixed background suggested that UCP2-deficient mice have higher serum insulin levels [10], it was recently shown that UCP2-deficient pancreatic β cells have reduced GSIS [17]. Our data further support this finding, as we have found normal serum insulin levels in KO mice (Figure 2C). Consistent with this, glucose tolerance was similar in all different groups, although DM animals started with lower fasting glucose levels (Figure 2D). Similarly, insulin sensitivity was comparable between all groups of animals (Figure 2E). Hence, our data indicate that UCP2 depletion in mtDNA mutator mice does not significantly change systemic glucose metabolism.

Analyses of other metabolic markers revealed that mtDNA mutator and DM mice show similar changes in the levels of different hormones involved in energy balance. We detected decreased levels of serum leptin, a central satiety agent (Figure 2F), while levels of ghrelin, a hunger-stimulating hormone were upregulated in both mtDNA mutator and DM mice (Figure 2G). It was previously shown that ghrelin acts by inducing UCP2-dependent changes of hypothalamic mitochondrial proliferation and respiration that are critical for the activation of different neurons involved in signaling of ghrelin-induced food intake [18]. Despite an increase in circulating ghrelin levels, we have not detected changes in UCP2 transcript levels in hypothalamus of mtDNA mutator mice (Figure 1D). It is possible that chronic induction of ghrelin does not affect UCP2 expression in hypothalamus, or the observed increase in circulating ghrelin levels is too low to induce this kind of change. Therefore, we believe that the changes induced by mitochondrial dysfunction do not affect the hypothalamic circuitry and feeding behaviour differently in mtDNA mutator and DM mice.

We have also found higher levels of glucagon and glucagon-like peptide 1 (GLP-1) (Figure 2H and 2I) that increase during prolonged periods of fasting and usually show inverted relation to serum insulin levels [19]. A synergism of low insulin and relative or absolute elevation of glucagon levels is viewed as a hormonal mechanism controlling the rate of hepatic substrate extraction for gluconeogenesis [19]. Finally, we also detected decreased levels of Resistin/RETN (Figure 2L), an adipose-secreted hormone linked to obesity and insulin resistance in rodents [20]. It was previously shown that the Resistin expression from adipose tissue and its serum levels are reduced in fasted mice [20]. We have not observed changes in the level of glucose-dependent insulinotropic polypeptide (GIP) (Figure 2J), indicating normal intestinal nutrient absorption [21], or plasminogen activator inhibitor-1 (PAI-1) (Figure 2K) that is linked to glucose intolerance and inflammation [22]. Taken together, these data show that mitochondrial dysfunction caused by increased mtDNA mutations induces a systemic fasting-like situation that is not significantly changed by UCP2 deficiency.

### Loss of UCP2 activates stress-induced markers and attenuates cardiac phenotypes in mtDNA mutator mice

**Figure 3:**
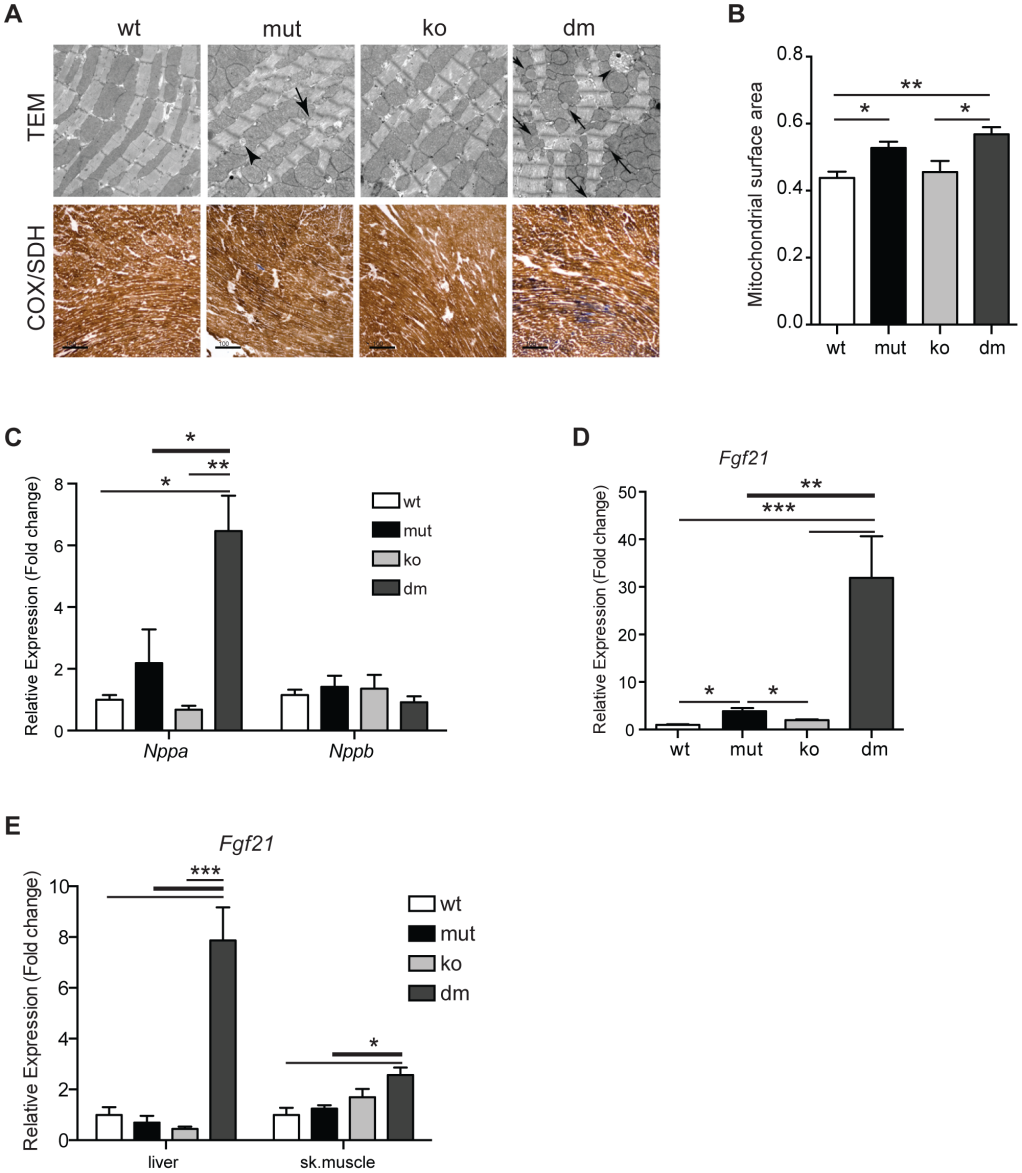
Characterization of mitochondrial cardiomyopathy in wild type (wt), mtDNA mutator (mut), UCP2-deficient wild type (ko) and mtDNA mutator (dm) mice. (A) Histological examination of cardiomyocytes from mut and dm mice. TEM – transmission electron micrographs. Arrows indicate lipid droplets, arrowheads abnormal mitochondria. COX/SDH - Enzyme histochemical staining for cytochrome *c* oxidase (COX) and succinate dehydrogenase (SDH) activities in heart. (B) Quantification of mitochondrial mass in transmission electron micrographs. (C) Relative expression levels of *Nppa* and *Nppb*, markers of heart failure. (D) Relative *Fgf21* mRNA expression levels in heart. (E) Relative *Fgf21* mRNA expression levels in liver and skeletal muscle. All analyses were performed on 25-week-old mice. Bars indicate mean level ± standard error of the mean (S.E.M.). Asterisks indicate level of statistical significance (*p<0.05 **p<0.01 ***p<0.001, Student's *t*-test).

Puzzled by our initial observation of decreased mean and maximal lifespan in DM mice, we proceeded to look more closely into changes caused by UCP2 depletion in heart, as this was the tissue with the strongest upregulation of UCP2 levels (Figure 1D) and dilated cardiomyopathy was recognized as one of the most prominent changes in mtDNA mutator mice [2]. Ultrastructural analyses of DM hearts revealed the accumulation of unusually shaped mitochondria and lipid droplets accompanied by an increase in mitochondrial mass already at 25 weeks of age (Figure 3A and 3B). We also observed a milder increase in mitochondrial mass in mtDNA mutator cardiomyocytes (Figure 3B). Upregulation of mitochondrial mass is a common compensatory mechanism opposing decreased mitochondrial function and is detected in different animal models and patients with mitochondrial diseases [23], [24]. Enzyme histochemical staining (COX-SDH) of heart sections revealed a higher incidence of mitochondrial deficiency in cardiomyocytes of DM mice at this age (Figure 3A). This suggests that high levels of UCP2 protect mtDNA mutator hearts from early respiratory deficiency and postpone pathological changes to a later age [2]. In agreement with this, we observed that levels of the natriuretic peptide precursor type A (*Nppa*), one of most commonly used marker of cardiac hypertrophy tripled in mtDNA mutator hearts after UCP2 depletion highlighting a progression of mitochondrial dysfunction in DM mice (Figure 3C).

Recently, it was shown that respiratory chain deficiency induces a mitochondrial stress response, marked by increased levels of FGF21 (Fibroblast growth factor 21), that directly correlate with the severity of mitochondrial dysfunction [25]. Expression of *Fgf21* in heart was also shown to have an important cardioprotective role [26]. Our latest results indicate that FGF21 could act as an initial signal that senses disrupted mitochondrial proteostasis in heart and activate different stress responses independent of respiratory chain deficiency [27]. Now, we observed an upregulation of *Fgf21* transcripts that was by an order of magnitude higher after UCP2 depletion in mtDNA mutator mice, signifying a more severe problem in DM hearts (Figure 3D). FGF21 is a cytokine that acts through autocrine or paracrine signaling and its main sources are thought to be liver, skeletal muscle and adipocytes [28]. Therefore, we also analyzed expression levels of *Fgf21* in liver and skeletal muscle. Mirroring the situation in heart, we found an upregulation of *Fgf21* levels in both liver and skeletal muscle exclusively in DM animals (Figure 3E). Upregulation of *Fgf21* levels in skeletal muscle is also detected in mtDNA mutator mice, but not before 37–40 weeks of age [29]. These results further support our conclusion that mtDNA mutator animals are under higher stress upon UCP2 depletion.

We also analysed cardiac function of mtDNA mutator mice before and after UCP2 depletion *in vivo* by high-resolution MRI (magnetic resonance imaging) in 18- to 20-week-old mice. Although we observed only mild changes in functional cardiac parameters, they were prevalent in DM mice (Figure S2).

### UCP2 depletion does not affect oxidative stress nor increases proton leak in mtDNA mutator hearts

Next we characterized the respiratory chain function in hearts of DM mice because of the findings of focal cytochrome *c* oxidase deficiency and increased mitochondrial mass in some cardiomyocytes (Figure 3). Our results show an additional decrease in complex I and IV respiratory chain enzyme activities in DM mice (Figure S3A). Even activity of complex II, the only mitochondrial respiratory chain (MRC) complex not affected in mtDNA mutator mice, was decreased, suggesting the existence of a general (toxic) effect of UCP2 deficiency on all respiratory chain complexes (Figure S3A). This was accompanied by a further 12–20% decrease in the mitochondrial ATP production rates (MAPR) in DM heart mitochondria incubated with different substrates (Figure S3B).

**Figure 4:**
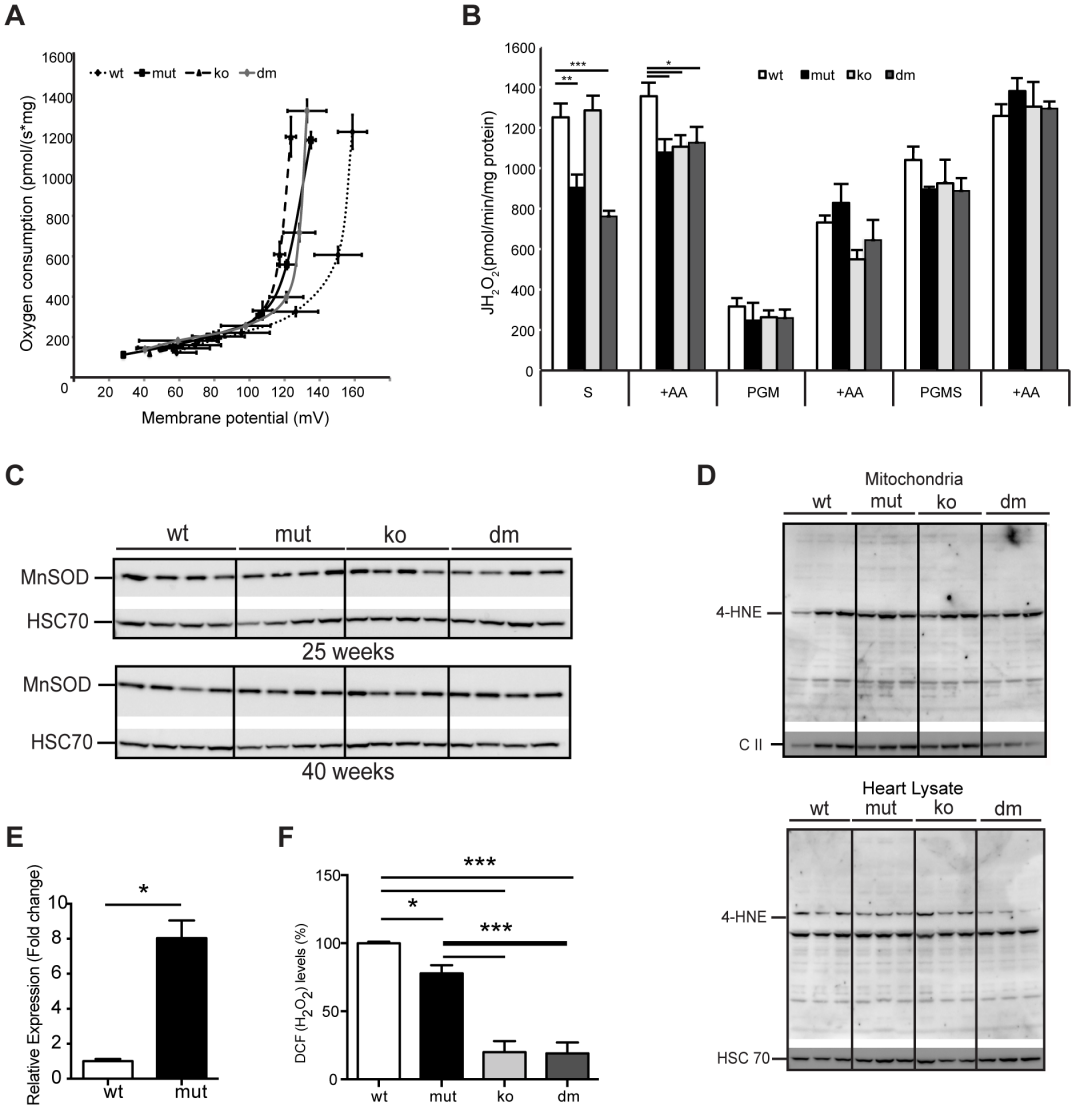
UCP2 deficiency does not affect proton leak kinetics or ROS production. (A) Flow-force relationship in heart mitochondria of 25-week-old wild type (wt), mtDNA mutator (mut), UCP2-deficient wild type (ko) and mtDNA mutator (dm) mice examined in the presence of succinate and increasing amounts of malonate (n = 6). Data points indicate mean levels ± standard error of the mean (S.E.M.). (B) Hydrogen peroxide production rate in heart mitochondria in the presence of succinate (S); Pyruvate-Glutamate-Malate (PGM) or Pyruvate-Glutamate-Malate-Succinate (PGMS) as substrates. Measurement in the presence of succinate alone detects reverse flow ROS production from both Complex I and Complex III. When mitochondria are incubated in the presence of pyruvate-glutamate-malate (PGM), the ROS production mainly originates from complex III and therefore it is usually lower. Treatment with Antimycin A (+AA), an inhibitor of CO III, was used as a positive control, to further induce ROS production (n = 4–5). Bars indicate mean level ± standard error of the mean (S.E.M.). (C) Western blot analyses of Manganese Superoxide Dismutase (MnSOD) in heart lysates of 25- and 40-week-old mice (n = 4). (D) Analysis of 4-Hydroxynonenal as measure for oxidative stress induced lipid peroxidation in isolated mitochondria (upper panel) and tissue lysates (lower panel) of 25-week-old mouse hearts. The mitochondrial Complex II 70 kDa protein (C II) was used as loading control in isolated mitochondria and the Heat shock 70 kDa protein 8 (HSC 70) in heart tissue lysates (n = 4). (E) Fold change of *Ucp2* transcript levels in primary MEFs (P1–P3). (F) ROS production in intact cells was assessed by flow cytometric analyses of primary (passage 1–3) mouse embryonic fibroblasts (MEFs) stained with CM-H_2_DCFDA that upon oxidation by ROS, particularly hydrogen peroxide (H_2_O_2_) and the hydroxyl radical (·OH), yields the fluorescent DCF product. Data are expressed as median values of fluorescence intensity ± standard error of the mean (S.E.M.). Asterisks indicate level of statistical significance (*p<0.05 **p<0.01 ***p<0.001, Student's *t*-test).

In order to understand why the loss of UCP2 leads to higher respiratory deficiency in mtDNA mutator mice, we examined the proton leak kinetics in heart mitochondria (Figure 4A). Our analysis showed that both resting oxygen consumption and membrane potential were normal in mtDNA mutator mice regardless of the presence of UCP2 (Figure 4A), indicating that UCP2 or increased levels of mtDNA mutations [2] do not affect proton leak kinetics in heart mitochondria of mtDNA mutator mice.

Mild uncoupling was proposed to significantly decrease ROS production in mitochondria [4]. Our initial hypothesis was that an eminent UCP2 overexpression has a protective role against a deleterious increase in ROS production. Therefore, we next determined net H_2_O_2_ production in isolated mitochondria. MtDNA mutator mitochondria, regardless of the presence or absence of UCP2, produced significantly less ROS than the wild type, when energized by succinate, and normal ROS levels, when we used mixed substrates that allow a maximal rate of mitochondrial respiration (Figure 4B). Increased reactive oxygen species (ROS) production often leads to compensatory upregulation of antioxidant responses in the cell. However, we found no evidence for the activation of oxidative stress responses, measured by the expression levels of different mitochondrial ROS scavenging enzymes (Figure S4A) and SOD2 protein levels (Figure 4C) in mtDNA mutator or DM mice at 25 and 40 weeks of age, consistent with our previous findings [3]. In addition, we did not detect increased oxidative stress in mtDNA mutator animals regardless of the presence of UCP2, as shown by normal levels of protein carbonyls (Figure S4B) and 4-hydroxy-2-nonenal (4-HNE) (Figure 4D). The ROS production in intact cells was measured in mouse embryonic fibroblasts that presented a high increase in *Ucp2* levels (Figure 4E), as our multiple efforts to isolate adult cardiomyocytes, especially from mtDNA mutator and DM animals, failed. We found a small decrease in ROS production in mtDNA mutator MEFs and a much stronger reduction in both UCP2 KO and DM cells (Figure 4F). Taken together, these results strongly indicate that UCP2 depletion in mtDNA mutator mice does not lead to an increase in ROS production or oxidative damage, and UCP2 upregulation is not a compensatory mechanism against increased oxidative stress.

### Loss of UCP2 decreases fatty acid oxidation in mtDNA mutator heart mitochondria

**Figure 5:**
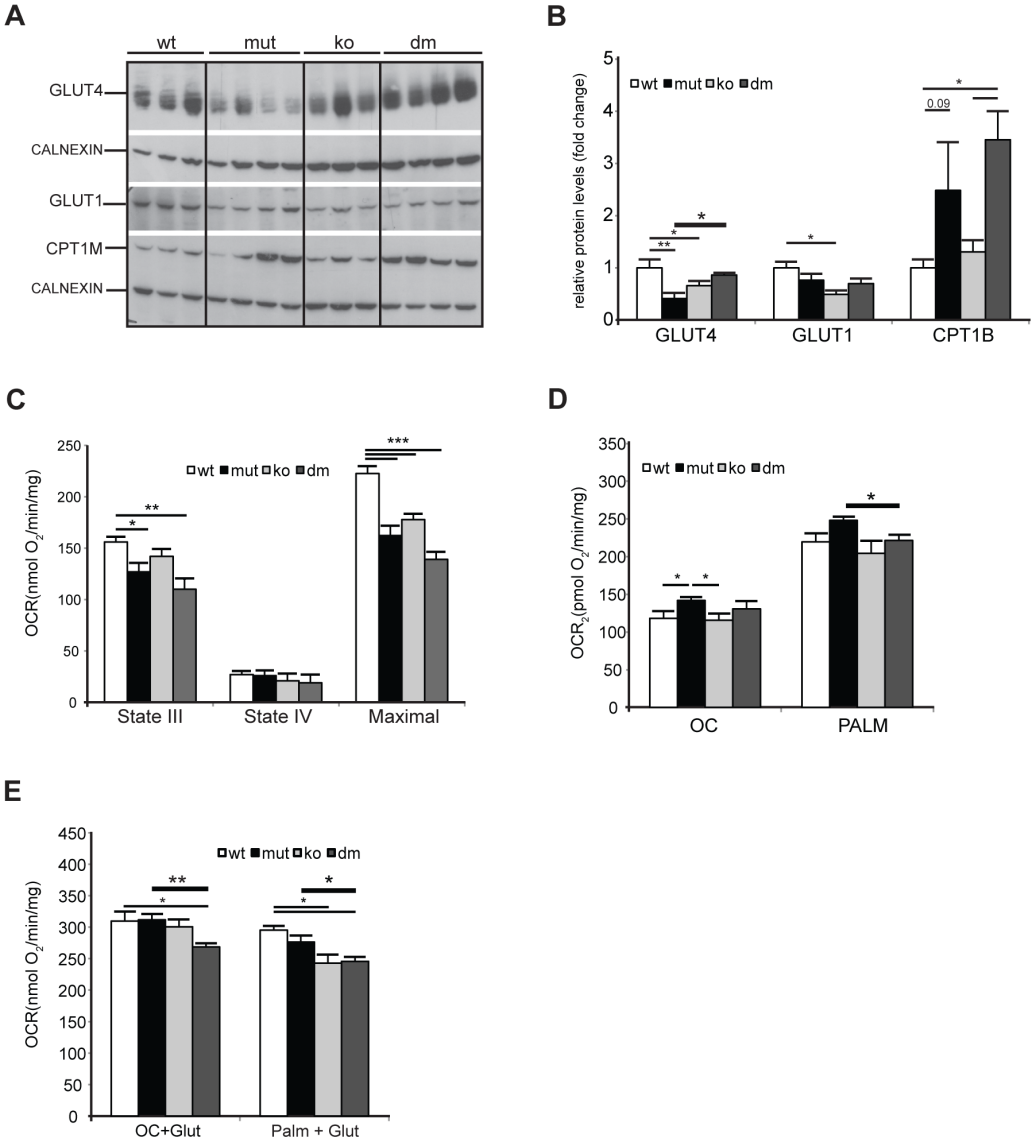
UCP2 promotes fatty acid oxidation in mtDNA mutator mitochondria. (A) Steady-state levels of mitochondrial glucose and fatty acid transporters: insulin-regulated glucose transporter (GLUT4), basal glucose transporter 1 (GLUT1) and mitochondrial carnitine palmitoyltransferase I (CPT1B) in wild type (wt), mtDNA mutator (mut), UCP2-deficient wild type (ko) and mtDNA mutator (dm) mice. Cytoplasmic calnexin was used as loading control. (B) Quantification of Western blots from (A). (C) Oxygen consumption rates in intact mitochondria in presence of pyruvate-glutamate-malate as substrates. State III (substrates+ADP); State IV (+oligomycin); MAX (+CCCP). (D) Oxygen consumption rates in intact mitochondria in the presence of medium (OC - octanoyl-carnitine) or long chain (PALM - palmitoyl-carnitine) fatty acids as substrates. (E) Maximal oxygen consumption rates upon addition of octanoyl-carnitine+glutamate (OC+Glut) or palmitoyl-carnitine+glutamate (PALM+Glut) to intact mitochondria. (n = 4–6). Bars indicate mean levels ± standard error of the mean (S.E.M.). Statistically significant differences between mut and dm are presented with thick lines. Asterisks indicate level of statistical significance (*p<0.05 **p<0.01 ***p<0.001, Student's *t*-test).

We next looked for an alternative role that UCP2 could play in mtDNA mutator hearts. Under normal conditions, the heart generates ATP by the consumption of energy substrates, mainly fatty acids (roughly 70%) with glucose and lactate contributing to the rest [30]. The rate of mitochondrial FAO is dependent on the level of fatty acids transported into mitochondria by Carnitine Palmitoyl Transferase 1 (muscle) - CPT1B, a mitochondrial enzyme associated with the outer mitochondrial membrane that mediates the transport of long-chain fatty acids across the membrane by binding them to carnitine. We detected a two to threefold increase in CPT1B levels in both mtDNA mutator and DM hearts pointing to an increased FFA uptake into heart mitochondria of both mutants (Figure 5A–B). The levels of the insulin-regulated glucose transporter, GLUT4, was two times lower in mtDNA mutator hearts, and was normalized upon UCP2 depletion (Figure 5A–B). The levels of GLUT1, a constitutively expressed glucose transporter, were not changed in mtDNA mutator or DM mitochondria (Figure 5A–B). These results demonstrate that mitochondrial dysfunction in mtDNA mutator mice increases the FFA uptake into heart mitochondria, while decreasing glucose uptake. Simultaneously, UCP2 upregulation allows higher or more efficient fatty acid oxidation. In the case of UCP2 deficiency combined with mitochondrial dysfunction, increased fatty acid uptake results in increased lipid accumulation inside cardiomyocytes, as observed in DM mice (Figure 3A). The defect in lipid handling in DM mice was further supported by changes in the expression of several genes involved in FFA transport and FAO in mitochondria like: *Cpt2* (Carnitine palmitoyl transferase 2), *Cact* (Carnitine-acylcarnitine translocase), *Acot10* (Acyl-CoA thioesterase 10) and *Lpl* (Lipoprotein lipase) (Figure S5A). Additionally, we observed an increase in the levels of *Fatp1* (Long-chain fatty acid transport protein 1) and *Fabp3* (Fatty acid binding protein 3, muscle), proteins that are involved in the cellular uptake of long-chain fatty acids [31], [32], only in DM hearts (Figure S5A).

We next looked at the expression of genes involved in glycolysis and found an increase in *Pdk4* (pyruvate dehydrogenase kinase, isozyme 4) levels in mtDNA mutator hearts that was highly augmented upon UCP2 depletion (Figure S5B). PDK4 is an enzyme that inactivates the pyruvate dehydrogenase complex (PDC) and therefore prevents the usage of pyruvate. This is crucial for the conservation of 3-C compounds for glucose synthesis when glucose is scarce [33] and could be a consequence of the high lactic acidosis observed in DM mice (Figure 2A).

To dissect the mechanism by which UCP2 regulates the metabolism of mtDNA mutator mice, we examined the metabolic capacity of isolated mitochondria using different substrates in the presence of ADP (State III): complex I (glutamate/malate) or fatty acids (octanoyl-carnitine or palmitoyl-carnitine) (Figure 5C and 5D, respectively). We also measured State IV, which represents the oxygen consumption not linked to ATP synthesis, i.e., respiration due to uncoupling (Figure 5C). In the presence of complex I substrates, oxygen consumption rates (OCR) in both inducible states (State III and maximal respiration) were decreased in mtDNA mutator and DM mice (Figure 5C). The respiratory control ratio (RCR), an index of mitochondrial uncoupling, was not changed in mtDNA mutator and DM heart mitochondria indicating that mitochondrial dysfunction did not trigger a higher proton leak, regardless of the presence of UCP2. When either medium (octanoyl-carnitine) or long chain fatty acids (palmitoyl-carnitine) were used as substrates, OCR was higher in mtDNA mutator mitochondria (Figure 5D). In agreement with these results, we observed decreased maximal oxidation rates in DM animals, when a mixture of glutamate plus either fatty acid was provided as a substrate for energy production (Figure 5E). In contrast, mtDNA mutator mitochondria had unchanged maximal OCR (Figure 5E), suggesting that, by increasing mitochondrial fatty acid oxidation, these animals can sustain normal energy production for prolonged periods and therefore postpone the onset of mitochondrial cardiomyopathy.

## Discussion

We describe a novel role of UCP2 in protection against early pathological changes in cardiomyocytes induced by respiratory deficiency due to increased mtDNA mutation load. Our results argue that this protective mechanism does not rely on the uncoupling activity of UCP2 leading to decreased ROS production in heart mitochondria. Instead, we show that UCP2 promotes fatty acid oxidation in heart, while also protecting from lactic acidosis resulting from systemic respiratory deficiency. This has an overall beneficial effect resulting in prolonged survival,delayed signs of mitochondrial cardiomyopathy and longer lifespan of mtDNA mutator mice.

Whether UCP2 plays a role in proton transport is still a matter of controversy. The increase in mitochondrial membrane potential in both macrophages and pancreatic β cells of UCP2 deficient mice is consistent with the proposed uncoupling activity [10], [34]. Besides, several lines of evidence showed that UCP2 decreases ROS production by lowering the membrane potential and therefore reducing reverse electron transfer into complex I [10], [34]. There are also strong arguments against the role of UCP2 in proton conductance. Unlike UCP1-deficient mice, UCP2 KO mice are resistant to cold exposure and they are not prone to obesity, even when fed a high fat diet, arguing against a role of UCP2 in energy expenditure [16]. Furthermore, depletion of UCP2 in tissues such as spleen or lung that express high levels of the protein does not change the uncoupling state of these cells [13]. In addition, a switch from fatty acid oxidation to glucose metabolism was demonstrated in UCP2-deficient mouse embryonic fibroblasts [35], in agreement with our results.

Until very recently no UCP2 substrates were known and it was even proposed that UCP2, like UCP3, act as an outward transporter of long-chain fatty acid anions from the mitochondrial matrix in situations where the fatty acid delivery to mitochondria exceeds the oxidative capacity [36]. However, a recent study showed that UCP2 acts as a metabolite transporter that regulates substrate oxidation in mitochondria [37]. UCP2 seems to be involved in both glucose and glutamine metabolism by catalyzing the exchange of malate, oxaloacetate, aspartate and malonate for phosphate plus a proton from opposite sides of the membrane [37]. The higher levels of citric acid cycle intermediates found in the mitochondria of cells where *Ucp2* is silenced, indicate that, by exporting C4 compounds out of mitochondria, UCP2 limits the oxidation of acetyl-CoA–producing substrates such as glucose and enhances glutaminolysis [37] Thus, UCP2 activity decreases the contribution of glucose to mitochondrial oxidative metabolism and promotes oxidation of alternative substrates such as glutamine and fatty acids [37].

We believe that UCP2 plays a role in stimulating lipid metabolism in mtDNA mutator cardiomyocytes. In combination with the observed general fasting-like phenotype that should promote increased lipolysis and availability of circulating fatty acids, this would allow better utilization of fatty acid oxidation while maintaining the energy-balance in conditions of moderate respiratory deficiency. As a result, high energy demanding tissues, such as heart, manage to produce enough energy, at least until fat stores are depleted. In the case of UCP2 deficiency, cells turn to glucose metabolism. However, respiratory deficiency makes the aerobic glycolysis inefficient, triggering a “vicious cycle” of metabolic events leading to diminishing efficiency of energy production, earlier development of mitochondrial cardiomyopathy and premature death. The increase in fatty acid delivery, in combination with defective fatty acid utilization, promotes lipid accumulation in the cardiomyocytes, which could additionally contribute to mitochondrial dysfunction in DM mice [38], [39]. Indeed, neurohumoral changes in heart failure, such as high adrenergic activity, can also increase the delivery of fatty acids to the heart by increasing lipolysis in adipose tissues [40]. Studies of substrate utilization in heart failure mostly show that fatty acid utilization is substantially decreased in advanced heart failure [41]. However, in contrast to patients with idiopathic dilated cardiomyopathy (DCM) and ischemic heart disease (IHD), patients with mitochondrial cardiomyopathy (MIC) have increased expression of genes involved in fatty acid metabolism, including *Ucp2, Cpt1, Pparα* and *Pgc1-α* [23]. It was debated that this is a maladaptive mechanism leading to the worsening of phenotypes [23]. Indeed, it may seem paradoxical that in mitochondrial cardiomyopathy, fatty acid metabolism is upregulated. However, our results argue that higher level of fatty acid oxidation is actually beneficial for the respiratory-deficient heart that cannot use aerobic glycolysis to its fullest and therefore probably fails to provide enough energy to sustain cardiac function.

Lactic acidosis, as detected in DM mice, is a common symptom in patients with mitochondrial diseases and is largely postponed in mtDNA mutator mice as a consequence of UCP2 overexpression [42]. Aerobic glycolysis normally does not result in an increase in circulating lactate levels, since tissues including heart can use lactate as energy source when their respiratory chain is intact [42]. However, systemic lactic acidosis could cause further decline of mitochondrial function in tissues, as shown in *Trans*-mitochondrial mice carrying high levels of pathogenic mtDNA molecules [43]. Therefore, we propose that the observed further decline of cardiac mitochondrial function in DM mice is a result of both tissue autonomous (respiratory deficiency combined with lipotoxicity) and systemic (lactic acidosis) factors.

Our study provides further evidence that premature aging phenotypes and mitochondrial dysfunction caused by accumulation of random mtDNA mutations arise without increased ROS production, thus strengthening our view that mechanisms other than oxidative stress play an important role in this process. However, it is still possible that aberrant ROS signalling or altered redox status might play a role in the development of different phenotypes observed in mtDNA mutator mice. This was supported by results showing that both, neuronal stem cell and hematopoietic pluripotent cell defects could be ameliorated by N-acetyl cysteine treatment (NAC - a compound with antioxidant capacity and an effect on redox balance). Furthermore, although no evidence of increased oxidative damage to proteins, lipids, or nucleic acids was ever found in the tissues or cells of mtDNA mutator mice [2], [44] their cardiomyopathy was attenuated by overexpression of mitochondrial-targeted catalase [45]. Hence, we believe that the interplay of mitochondrial dysfunction and ROS signaling is likely much more complex than we currently understand and mtDNA mutator is an invaluable model to dissect this even further. However, our results argue that UCP2 protein does not play a significant role in this process, or at least not in the highly energy demanding tissue such as heart.

## Materials and Methods

### Animals

UCP2-deficient mice and mtDNA mutator animals used in this study have been backcrossed for more than 20 generations to C57Bl6 background. To produce the double mutant (DM), *PolgA^mut/mut^; Ucp2^−/−^* animals, we initially crossed mice heterozygous for the mtDNA mutator allele (*+/PolgA^mut^*) with mice deficient in UCP2 (*Ucp2^−/−^*) [2], [34]. After a series of mating steps, we obtained UCP2-deficient mice that also carry one copy of the mtDNA mutator allele (*+/PolgA^mut^; Ucp2^−/−^*). These were then intercrossed to obtain UCP2 KO and DM mice. Wild type and mtDNA mutator mice were generated by intercrossing animals heterozygous for the mtDNA mutator allele [2]. Genotyping for both alleles was performed as described earlier [2], [34]. Mice were group-housed with food and water *ad libitum* and were maintained on a 12 h light-dark cycle. Animal protocols were in accordance with guidelines for humane treatment of animals and were reviewed and approved by the Animal Ethics Committee of the Stockholm region, Sweden and North Rhine-Westphalia, Germany.

### Food intake, glucose and lactate measurements

Daily food intake was calculated as the average intake of normal chow diet during 2 weeks. Mice were acclimated to the food intake settings for 5 days and were weighed every week to follow changes in body weight. Fed blood glucose and lactate concentrations were measured after tail-vein incision in 25-week-old animals, using glucose or lactate strips, which were read for absorbance in a reflectance meter (ACCU-CHEK AVIVA and Accutrend Plus, Roche Diagnostics GmBH, Mannheim, Germany).

### Serum free fatty acids

Serum Non-esterified fatty acids (NEFA) levels were determined using an acyl-CoA oxidase based colorimetric kit (WAKO NEFA–C; WAKO Wako Life Sciences, Inc., USA). NEFA standard solutions were used for the linear regression plot and absorbency measured at 550 nm in a Paradigm plate reader (Molecular Devices).

### Western and northern blot analyses

Protein lysates were obtained by disrupting the tissue in lysis buffer (50 mM HEPES, pH 7.4, 1% Triton X-100, 0.1 M NaF, 10 mM Na Orthovanadat, 10 mM EDTA, 0.1% SDS, 50 mM NaCl, 20 mM PMSF, 1 tablet protease inhibitor (Roche) in a tissue homogenizer (Precellys24, Bertin Technologies). After centrifugation, supernatants, containing solubilised proteins, were used for further analysis. Mitochondria were isolated from different tissues as previously described [46]. The antibodies and dilutions used for Western blot analyses are as follows: CALNEXIN (1∶2000, Calbiochem), Complex II 70 kDA Fp subunit (1∶1000, Molecular Probes), CPT1B (1∶1000, alpha Diagnostic), GLUT-1 (1∶1000, Abcam), GLUT-4 (1∶1000, Millipore), HSC-70 (1∶10000, Santa Cruz), MnSOD (1∶1000, Upstate Millipore), TOM20 (1∶1000, Santa Cruz), UCP3 (1∶1000, Abcam), UCP2 (1∶500, [12]), 4-HNE (1∶3000, Millipore). Oxyblots were performed according to the manufacturer instructions with 15–20 µg proteins (OxyBlot Protein Oxidation Detection Kit, Millipore). Northern blot analyses were performed as described [47].

### Measurement of Δp by radiolabeled probes distribution

Measurements were performed in respiration buffer containing 120 mM Sucrose, 50 mM KCl, 20 mM Tris, 1 mM EGTA, 4 mM KH_2_PO_4_, 2 mM MgCl_2_*6 H_2_O, 0.1% fatty acid free BSA, pH 7,2. Matrix space was determined by using 4.5 mCi [^3^H]H_2_O and 0.45 mCi inner membrane impermeable [^14^C]sucrose. Δ*Ψ* and ΔpH were determined by the distribution of [^3^H]TPMP^+^ and [^3^H]acetate, respectively. [^3^H]TPMP^+^ is a lipophilic cation and its binding coefficient was determined as being equal to 0.38 [48]. Routinely, after equilibration, mitochondria were separated from the medium by rapid centrifugation (12000 g, 30 s), then treated as described previously [49].

### Quantitative real-time PCR

Isolated RNA was treated with DNAse (DNA-free Kit, Ambion) and subsequently reversely transcribed with the High capacity reverse transcription kit (Applied Biosystems). Probes for target genes were from TaqMan Assay-on-Demand kits (Applied Biosystems) (*Cat, Cpt1a, Cpt2, Fgf21, Glut4, Gpx, Hk1, Lpl, Sod1, Sod2, Txn1, Txn2, Ucp2*). For other genes Brilliant III Ultra-Fast SYBR Green QPCR Master Mix (Agilent Technologies) and primers as in Table S2 were used. Samples were adjusted for total RNA content by TATA box binding protein (*Tbp*) and Hypoxanthine-guanine phosphoribosyltransferase (*Hprt*).

### Enzyme histochemistry and respiratory chain function

Enzyme histochemical analyses of succinate dehydrogenase (SDH) and cytochrome c oxidase (COX) activities were performed on 14 µm cryostat sections of fresh frozen hearts [50]. The measurement of respiratory chain enzyme complex activities was performed as previously described [51].

### Levels of serum hormones

Levels of hormones were quantified in serums of 30-week-old mice (diluted 1∶4) using Magnetic Bead Metabolic Assays (Bio-Plex, Bio-Rad, UK) and a Bio-Plex 200 system (Bio-Rad, UK) according to the manufacturer's instructions.

### Transmission electron microscopy

Small pieces from the myocardium were fixed in 2% glutaraldehyde and 1% paraformaldehyde in 0.1 M phosphate buffer (PB), pH 7.4 at room temperature and stored at 4°C. Specimens were rinsed in a PB and postfixed in 2% osmiumtetroxide in PB at 4°C for 2 h, dehydrated in ethanol followed by acetone and embedded in LX-112 (Ladd, Burlington, USA). Ultrathin sections (approximately 40–50 nm) were cut by a Leica ultracut (Leica, Wien, Austria). Sections were contrasted with uranyl acetate followed by lead citrate and examined in a Tecnai 10 transmission electron microscope (Fei Company, Eindhoven, The Netherlands) at 100 kV. Digital images were randomly taken by using a Veleta camera (Olympus Soft Imaging Solutions, GmbH, Münster, Germany) on myofibrils from sections of the myocardium.

### Proton leak measurements

Mitochondria were isolated from heart tissue according to [46] and resuspended in MSE buffer (225 mM Mannitol, 75 mM Sucrose, 1 mM EGTA, 5 mM HEPES) at a final concentration of 20 µg/ul. The mitochondrial membrane potential was assessed by fluorimetric detection of Rhodamine 123 fluorescence at a Shimadzu 5003PC spectrofluorimeter. Calibration curves to determine membrane potential from K^+^-diffusion potentials were performed with 140 µg Rhodamine 123 stained mitochondria treated with 1 µg/ml valinomycin, 2 µg/ml oligomycin and 100 nM rotenone in oxygen-saturated potassium-free buffer (10 mM Na H_2_PO_4_, 60 mM NaCl, 60 mM Tris/HCl, 110 mM Mannitol, 0.5 mM EDTA, pH 7.4) containing 5 mM glutamate and 5 mM malate by titration with increasing KCl concentrations.

Measurement of membrane potential was conducted with 140 µg Rhodamine 123 stained mitochondria in air-saturated potassium-containing buffer (10 mM K H_2_PO_4_, 60 mM KCl, 60 mM Tris/HCl, 110 mM Mannitol, 0.5 mM EDTA, pH 7.4) supplemented with 10 mM succinate by addition of increasing concentrations of malonate. Total uncoupling was assessed by addition of 1 µM TTFB. Oxygen consumption was analyzed under the conditions described for the determination of membrane potential in air-saturated potassium-containing buffer at 30°C in an Oroboros oxygraph.

### Respiration measurements on fatty acids

The respiration measurements were performed on isolated heart mitochondria (as previously described) at 30°C using a high-resolution Oroboros-oxygraph in air-saturated medium consisting of 110 mM mannitol, 60 mM KCl, 5 mM MgCl_2_, 10 mM KH_2_PO_4_, 0.5 mM Na_2_EDTA, and 60 mMTris – HCl (pH 7.4) containing either 5 mM malate, 2 mM ADP, 1 mM octanoyl carnitine or 5 mM malate, 2 mM ADP, 50 uM palmitoyl carnitine. The maximal oxidation rates in these conditions were analyzed by addition of 10 mM glutamate. The quality of mitochondria was assessed by incubation with 0.2 mM atractyloside.

### Mitochondrial respiration and hydrogen peroxide production rate

Mitochondrial oxygen consumption flux was measured as previously described [52] at 37°C using 65–125 µg of crude mitochondria diluted in 2.1 ml of mitochondrial respiration buffer (120 mM sucrose, 50 mM KCl, 20 mM Tris-HCl, 4 mM KH_2_PO_4_, 2 mM MgCl_2_, 1 mM EGTA, pH 7.2) in an Oxygraph-2 k (OROBOROS INSTRUMENTS, Innsbruck, Austria). The oxygen consumption rate was measured using, either 10 mM pyruvate, 5 mM glutamate and 5 mM malate (PGM) or 10 mM succinate (S). Oxygen consumption was assessed in the phosphorylating state with 1 mM ADP (state 3) or non-phosphorylating state by adding 2.5 µg/ml oligomycin (pseudo state 4). In the control mitochondria, the respiratory control ratio (RCR) values were >8 with pyruvate/glutamate/malate. The rate of H_2_O_2_ production was determined by monitoring the oxidation of the fluorogenic indicator Amplex red (1 µM) in the presence of horseradish peroxidase (5 U/ml). Fluorescence was recorded at the following wavelengths: excitation 560 nm and emission 590 nm. A standard curve was obtained by adding known amounts of H_2_O_2_ to the assay medium in the presence of the reactants. Mitochondria (65 µg protein/ml) were incubated in the respiratory medium at 37°C and the H_2_O_2_ production rate was initiated by adding either 10 mM pyruvate, 5 mM glutamate and 5 mM malate (PGM) or 10 mM succinate (S) or all substrates together (PGMS). Complex III inhibitor antimycin A was added (0.5 µM) after initial reading to further induce ROS production. The H_2_O_2_ production rate was determined from the slope of a plot of the fluorogenic indicator versus time.

### ROS detection in MEFs

Primary MEFs (passage 1-3) were cultivated in Dulbecco's modified Eagle's medium (DMEM) with high glucose (4500 mg/L), 4 mM L-glutamine and 1 mM sodium pyruvate supplemented with 10% FBS and 100 units/ml penicillin, and 100 µg of streptomycin.

Cells (80-90% confluence) were washed by warm PBS, harvested by trypsinization and collected with complete culture medium by centrifugation (5 min at 200 g). After washing with PBS, cells were resuspended in 500 µl PBS, stained with 10 µmol/L of CM-H2DCFDA (5-(and-6-)-carboxy-2′,7′-dichlorodihydrofluorescein diacetate) and incubated in a cell incubator [(37°C), high relative humidity (95%), and controlled CO2 level (5%)] in the dark for 45 min. Propidium Iodide (1 µg/ml) was added to gate the living cells and tubes were kept on ice for immediate flow cytometry analysis. A total of 25000 events were analyzed and data expressed as Median +− SEM.

### *In vivo* measurement of cardiac function

Magnetic resonance imaging (MRI) was performed using a vertical Bruker AVANCE^III^ 9.4 Tesla Wide Bore NMR spectrometer equipped with an actively shielded 57-mm gradient set and a 30-mm birdcage resonator. Mice were anesthetized with 1.5% isoflurane and kept at body temperature during the whole experiment. Acquisition and analysis of data were performed as described by Jacoby et al. [53].

### Indirect calorimetry and physical activity

All measurements were performed in a PhenoMaster System (TSE systems, Bad Homburg, Germany), which allows measurement of metabolic performance and activity monitoring by an infrared light-beam frame. Mice were placed at room temperature (22°C–24°C) in 7.1-l chambers of the PhenoMaster open circuit calorimetry. Mice were allowed to acclimatize in the chambers for at least 24 hr. Locomotor activity and parameters of indirect calorimetry were measured for at least 48 hr. Food and water were provided ad libitum.

### Glucose and insulin tolerance test

For the glucose tolerance test, animals were fasted for approximately 16 hours and blood glucose was measured immediately prior and 15, 30, 60, and 120 minutes after the intraperitoneal injection of a glucose solution (2 g/kg body weight).

Insulin tolerance tests were performed on random fed animals between 2:00 and 5:00 PM. Animals were injected with 0.75 U/kg body weight of human insulin (Insuman Rapid 40 IU/ml) into the peritoneal cavity. Blood glucose values were measured immediately before and 15, 30, and 60 min after the injection. Results were expressed as percentage of initial blood glucose concentration.

### Statistical analysis

Statistical significance for all figures was determined by Student's t-test. In addition we have analyzed all results with one-way ANOVA followed by Tukey's Multiple Comparison. Results of ANOVA analyses are presented in Table S1.

## Supporting Information

Figure S1 Metabolic phenotyping. (A) Food consumption. (B) Body weight. (C) Body composition. (D) Indirect calorimetry shows oxygen consumption (VO2), (E) Cage activity, (F) Energy expenditure and (G) Respiratory Exchange Ratio (RER) per hour during 24 h recording - of wild type (wt), mtDNA mutator (mut), UCP2-deficient wild type (ko) and mtDNA mutator mice (dm) mice at 20 weeks of age (n = 6). Bars indicate mean levels ± standard error of the mean (S.E.M.). Statistically significant differences between mut and dm are presented with thick lines. Asterisks indicate level of statistical significance (*p<0.05; **p<0.005; ***p<0.001, Student's *t*-test). Despite comparable food consumption in all animal groups, a significant reduction in body weight was observed in mtDNA mutator and DM mice. The observed decrease was mainly due to the loss of fat content in both groups. The oxygen consumption, energy expenditure and respiratory exchange ratio (RER) were higher during dark cycle (a period of high activity) in DM mice, suggesting that these mice had a preference toward using glucose for energy production.
(PDF)

Figure S2 *In vivo* analysis of cardiac function by high-resolution MRI. (A) Body weight. (B) Respiration rate. (C) Heart rate. (D) Myocardial mass. (E) End-diastolic volume (EDV) and end-systolic volume (ESV). (F) Ejection fraction. (G) Stroke volume. (H) Heart wall thickness. (I) Left ventricle to body weight ratio (LV/BW). (J) Mass of left ventricle. (K) cardiac output and (L) cardiac output to body weight ratio. (n = 6–9). Measurements were performed in -wild type (wt), mtDNA mutator (mut), UCP2-deficient wild type (ko) and mtDNA mutator mice (dm) at 18–20 weeks of age. Bars indicate mean levels ± standard error of the mean (S.E.M.). Statistically significant differences between mut and dm are presented with thick lines. Asterisks indicate level of statistical significance (*p<0.05; **p<0.005; ***p<0.001, Student's *t*-test). The analysis was limited to 18- to 20-week-old mice as both mtDNA mutator and DM mice had a pronounced decrease in respiration rates upon anaesthesia that often resulted in death of animals at older age. The observed changes were prevalent in DM mice as illustrated by decreased heart rate, cardiac output, cardiac output to body weight ratio and increased wall thickness, left ventricle size (LV), or left-ventricle-to-body-weight ratio (LV/WG). End-systolic volume (ESV) was higher in mtDNA mutator mice, but stroke volume (SV) was preserved, consistent with the known mechanisms of adaptation.
(PDF)

Figure S3 Biochemical analyses of respiratory chain function. (A) Relative activities of respiratory chain enzymes. Complex I (CO I) - NADH coenzyme Q reductase; Complex I+III (CO I/III) - NADH cytochrome *c* reductase; Complex II (CO II) – succinate dehydrogenase; Complex II+III (CO II/III) - succinate:cytochrome *c* reductase; Complex IV (CO IV) - cytochrome *c* oxidase (COX). (B) Measurements of mitochondrial ATP production rate (MAPR) per unit of CS activity with substrates that enter the respiratory chain at different points. MAPR was determined with seven different substrate combinations: glutamate + succinate (G+S), glutamate + malate (G+M), TMPD + ascorbate (T+A), pyruvate + malate (P+M), palmitoyl-L-carnitine + malate (PC+M), succinate + rotenone (S+R) and succinate (S) (n = 6). Measurements were performed on wild type (wt), mtDNA mutator (mut), UCP2-deficient wild type (ko) and mtDNA mutator mice (dm) mice at 20 weeks of age. Bars indicate mean levels ± standard error of the mean (S.E.M.). Statistically significant differences between mut and dm are presented with thick lines. Asterisks indicate level of statistical significance (*p<0.05; **p<0.005; ***p<0.001, Student's *t*-test).
(PDF)

Figure S4 Expression analysis of ROS scavenging enzymes and detection of oxidative damage. (A) Relative expression levels of antioxidant enzymes that are involved in (i) hydrogen-peroxide removal: Cat - catalase and Gpx1 - glutathione peroxidase; (ii) superoxide removal: Sod1 - copper zinc superoxide dismutase and Sod2 - mitochondrial manganese superoxide dismutase; (iii) redox balance: *Txn1* and *Txn2* - thioredoxin 1 and 2. (B) Analysis of oxidative stress-related carbonyl groups (Oxyblot) in isolated heart mitochondria. Measurements were performed on wild type (wt), mtDNA mutator (mut), UCP2-deficient wild type (ko) and mtDNA mutator mice (dm) mice at 25 weeks of age. The mitochondrial Complex II 70 kDa protein (Co II) and Ponceau S staining serve as loading controls. Bars indicate mean levels ± standard error of the mean (S.E.M.).
(PDF)

Figure S5 Expression analysis of genes involved in glycolysis and fatty acid oxidation-related genes. (A) Relative expression levels of lipolytic and FAO-related genes: *Acot10* - acyl- Coenzyme A thioesterase 3, mitochondrial; *Cpt1a* - carnitine palmitoyl transferase 1 (liver); *Cpt1b* - carnitine palmitoyl transferase 1 (muscle); *Cpt2* - carnitine palmitoyl transferase 2; *Cact* - Carnitine-acylcarnitine translocase; *Ckmt2* - creatine kinase, mitochondrial 2; *Mcad* - medium- chain acyl-CoA dehydrogenase; *Mte1* – mitochondrial thioesterase 1; *Fabp3* – fatty acid binding protein, muscle; *Fatp1* – Long-chain fatty acid transport protein 1; *Lpl* – lipoprotein lipase. (n = 5). (B) Relative expression levels of genes involved in glycolysis: *Glut1* - Glucose transporter 1, *Glut4* - Glucose transporter type 4; *Hk1* and *Hk2* - Hexokinase 1 and 2; *Pdha* - pyruvate dehydrogenase, subunit A; *Pdk4* - Pyruvate dehydrogenase kinase isozyme 4, mitochondrial. Bars indicate mean levels ± standard error of the mean (S.E.M.). Statistically significant differences between mut and dm are presented with thick lines. Asterisks indicate level of statistical significance (*p<0.05; **p<0.005; ***p<0.001, Student's *t*-test).
(PDF)

Table S1 Statistical analysis of results by one-way ANOVA analysis. Measurements were performed on wild type (wt), mtDNA mutator (mut), UCP2-deficient wild type (ko) and mtDNA mutator mice (dm) mice. Asterisks indicate level of statistical significance (*p<0.05; **p<0.005; ***p<0.001).
(XLS)

Table S2 List of primers used for quantitative real-time PCR analysis using Brilliant III Ultra-Fast SYBR Green QPCR Master Mix (Agilent Technologies).
(XLSX)
