# Supplementary figures and images for: Loss of UCP2 Attenuates Mitochondrial Dysfunction without Altering ROS Production and Uncoupling Activity
Source: PLoS Genet. 2014 Jun 19;10(6):e1004385. doi: 10.1371/journal.pgen.1004385 (PMC4063685; doi:10.1371/journal.pgen.1004385)

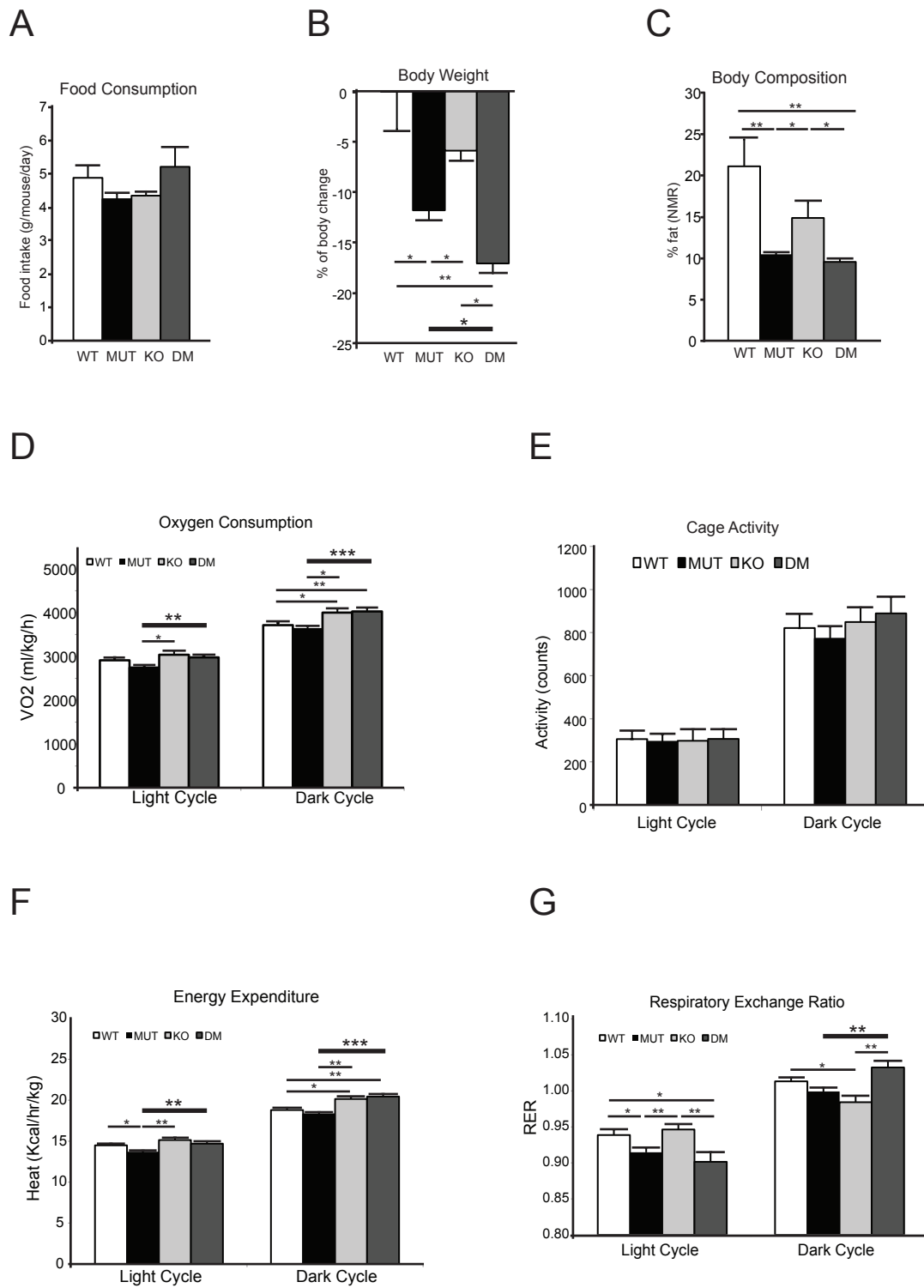

Kukat et al. Figure S1.

Supplement: Figure S1 — Metabolic phenotyping. (A) Food consumption. (B) Body weight. (C) Body composition. (D) Indirect calorimetry shows oxygen consumption (VO2), (E) Cage activity, (F) Energy expenditure and (G) Respiratory Exchange Ratio (RER) per hour during 24 h recording - of wild type (wt), mtDNA mutator (mut), UCP2-deficient wild type (ko) and mtDNA mutator mice (dm) mice at 20 weeks of age (n = 6). Bars indicate mean levels ± standard error of the mean (S.E.M.). Statistically significant differences between mut and dm are presented with thick lines. Asterisks indicate level of statistical significance (*p<0.05; **p<0.005; ***p<0.001, Student's t-test). Despite comparable food consumption in all animal groups, a significant reduction in body weight was observed in mtDNA mutator and DM mice. The observed decrease was mainly due to the loss of fat content in both groups. The oxygen consumption, energy expenditure and respiratory exchange ratio (RER) were higher during dark cycle (a period of high activity) in DM mice, suggesting that these mice had a preference toward using glucose for energy production. (PDF) [file pgen.1004385.s001.pdf]

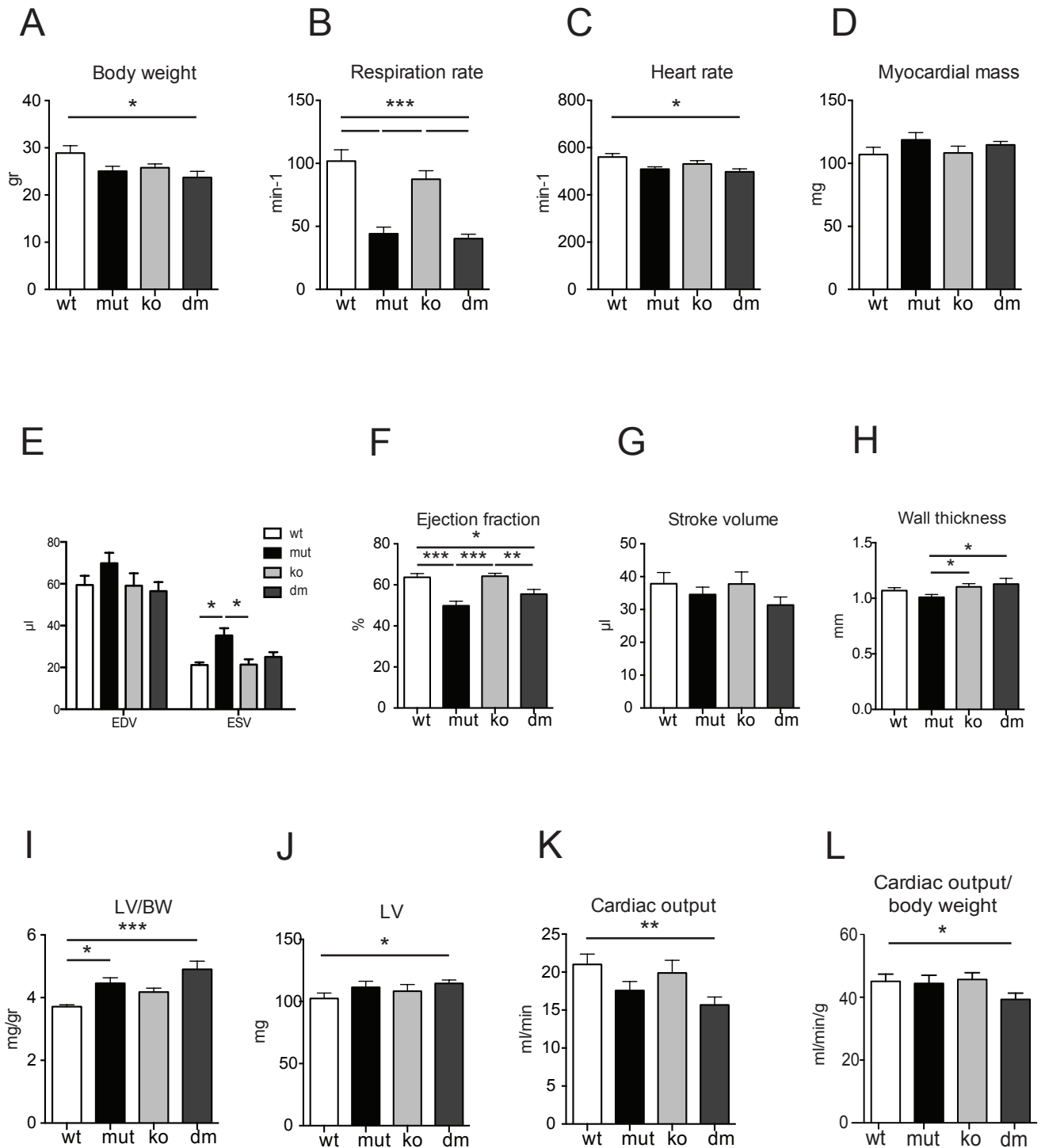

Kukat et al. Figure S2

Supplement: Figure S2 — In vivo analysis of cardiac function by high-resolution MRI. (A) Body weight. (B) Respiration rate. (C) Heart rate. (D) Myocardial mass. (E) End-diastolic volume (EDV) and end-systolic volume (ESV). (F) Ejection fraction. (G) Stroke volume. (H) Heart wall thickness. (I) Left ventricle to body weight ratio (LV/BW). (J) Mass of left ventricle. (K) cardiac output and (L) cardiac output to body weight ratio. (n = 6–9). Measurements were performed in -wild type (wt), mtDNA mutator (mut), UCP2-deficient wild type (ko) and mtDNA mutator mice (dm) at 18–20 weeks of age. Bars indicate mean levels ± standard error of the mean (S.E.M.). Statistically significant differences between mut and dm are presented with thick lines. Asterisks indicate level of statistical significance (*p<0.05; **p<0.005; ***p<0.001, Student's t-test). The analysis was limited to 18- to 20-week-old mice as both mtDNA mutator and DM mice had a pronounced decrease in respiration rates upon anaesthesia that often resulted in death of animals at older age. The observed changes were prevalent in DM mice as illustrated by decreased heart rate, cardiac output, cardiac output to body weight ratio and increased wall thickness, left ventricle size (LV), or left-ventricle-to-body-weight ratio (LV/WG). End-systolic volume (ESV) was higher in mtDNA mutator mice, but stroke volume (SV) was preserved, consistent with the known mechanisms of adaptation. (PDF) [file pgen.1004385.s002.pdf]

A

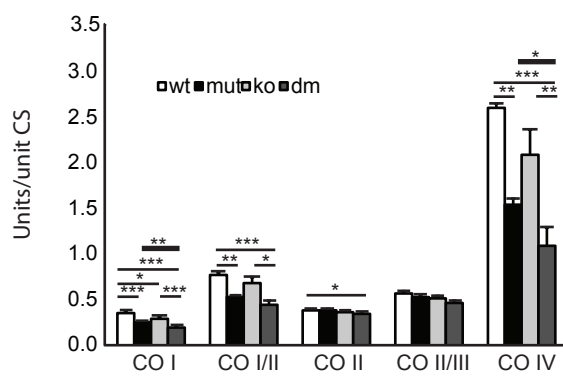

B

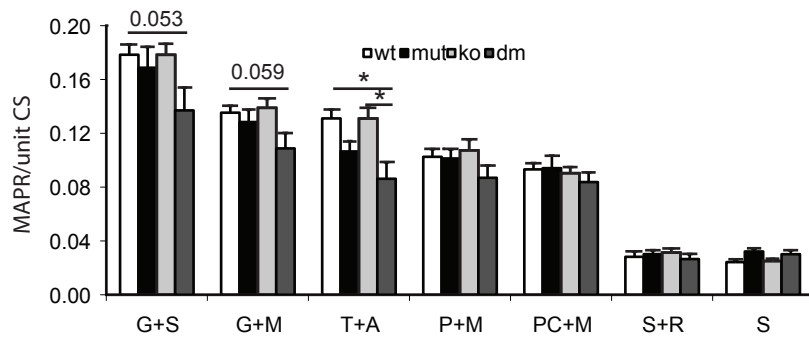

Supplement: Figure S3 — Biochemical analyses of respiratory chain function. (A) Relative activities of respiratory chain enzymes. Complex I (CO I) - NADH coenzyme Q reductase; Complex I+III (CO I/III) - NADH cytochrome c reductase; Complex II (CO II) – succinate dehydrogenase; Complex II+III (CO II/III) - succinate:cytochrome c reductase; Complex IV (CO IV) - cytochrome c oxidase (COX). (B) Measurements of mitochondrial ATP production rate (MAPR) per unit of CS activity with substrates that enter the respiratory chain at different points. MAPR was determined with seven different substrate combinations: glutamate + succinate (G+S), glutamate + malate (G+M), TMPD + ascorbate (T+A), pyruvate + malate (P+M), palmitoyl-L-carnitine + malate (PC+M), succinate + rotenone (S+R) and succinate (S) (n = 6). Measurements were performed on wild type (wt), mtDNA mutator (mut), UCP2-deficient wild type (ko) and mtDNA mutator mice (dm) mice at 20 weeks of age. Bars indicate mean levels ± standard error of the mean (S.E.M.). Statistically significant differences between mut and dm are presented with thick lines. Asterisks indicate level of statistical significance (*p<0.05; **p<0.005; ***p<0.001, Student's t-test). (PDF) [file pgen.1004385.s003.pdf]

A

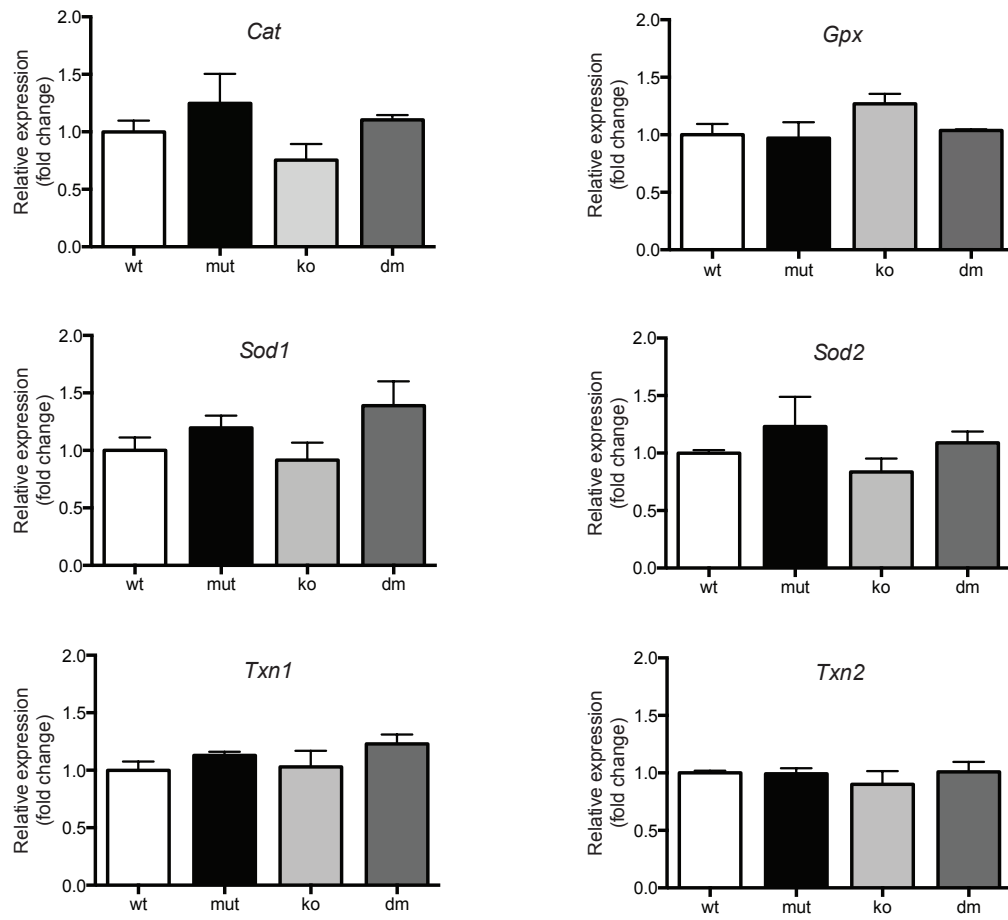

B

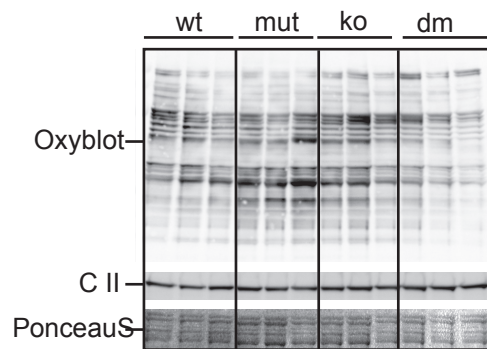

Kukat et al. Figure S4.

Supplement: Figure S4 — Expression analysis of ROS scavenging enzymes and detection of oxidative damage. (A) Relative expression levels of antioxidant enzymes that are involved in (i) hydrogen-peroxide removal: Cat - catalase and Gpx1 - glutathione peroxidase; (ii) superoxide removal: Sod1 - copper zinc superoxide dismutase and Sod2 - mitochondrial manganese superoxide dismutase; (iii) redox balance: Txn1 and Txn2 - thioredoxin 1 and 2. (B) Analysis of oxidative stress-related carbonyl groups (Oxyblot) in isolated heart mitochondria. Measurements were performed on wild type (wt), mtDNA mutator (mut), UCP2-deficient wild type (ko) and mtDNA mutator mice (dm) mice at 25 weeks of age. The mitochondrial Complex II 70 kDa protein (Co II) and Ponceau S staining serve as loading controls. Bars indicate mean levels ± standard error of the mean (S.E.M.). (PDF) [file pgen.1004385.s004.pdf]

A

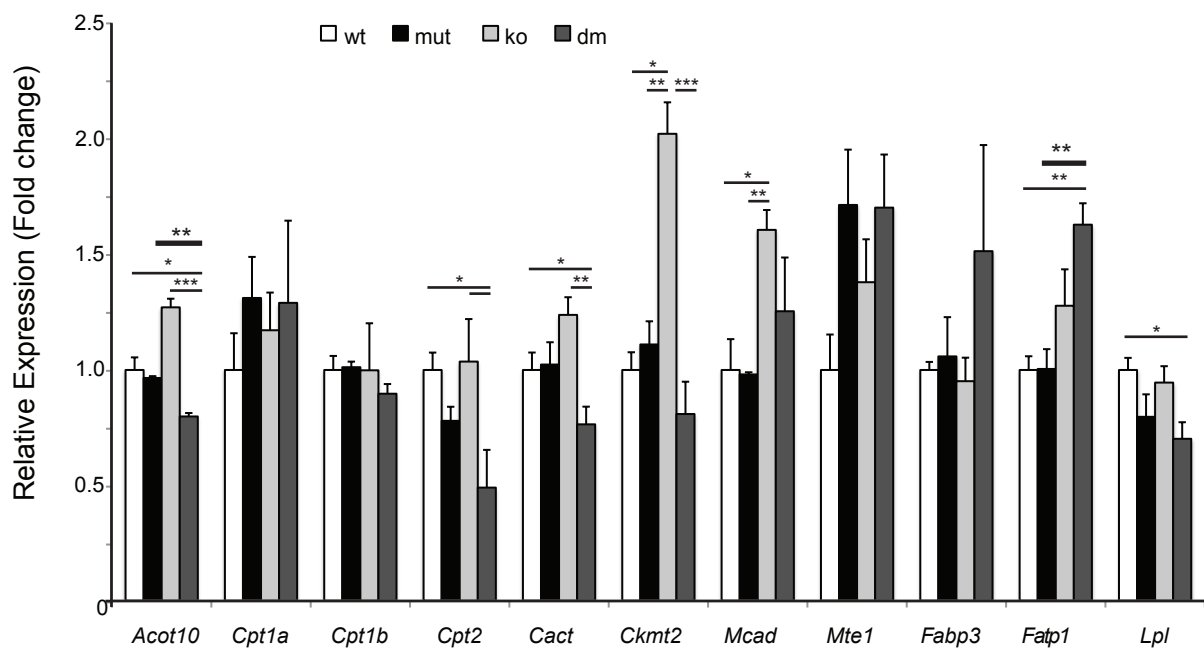

B

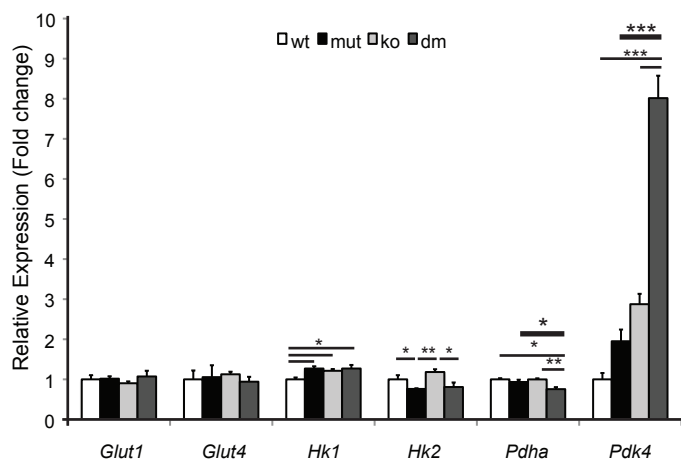

Supplement: Figure S5 — Expression analysis of genes involved in glycolysis and fatty acid oxidation-related genes. (A) Relative expression levels of lipolytic and FAO-related genes: Acot10 - acyl- Coenzyme A thioesterase 3, mitochondrial; Cpt1a - carnitine palmitoyl transferase 1 (liver); Cpt1b - carnitine palmitoyl transferase 1 (muscle); Cpt2 - carnitine palmitoyl transferase 2; Cact - Carnitine-acylcarnitine translocase; Ckmt2 - creatine kinase, mitochondrial 2; Mcad - medium- chain acyl-CoA dehydrogenase; Mte1 – mitochondrial thioesterase 1; Fabp3 – fatty acid binding protein, muscle; Fatp1 – Long-chain fatty acid transport protein 1; Lpl – lipoprotein lipase. (n = 5). (B) Relative expression levels of genes involved in glycolysis: Glut1 - Glucose transporter 1, Glut4 - Glucose transporter type 4; Hk1 and Hk2 - Hexokinase 1 and 2; Pdha - pyruvate dehydrogenase, subunit A; Pdk4 - Pyruvate dehydrogenase kinase isozyme 4, mitochondrial. Bars indicate mean levels ± standard error of the mean (S.E.M.). Statistically significant differences between mut and dm are presented with thick lines. Asterisks indicate level of statistical significance (*p<0.05; **p<0.005; ***p<0.001, Student's t-test). (PDF) [file pgen.1004385.s005.pdf]
